# Familial Multiple Telangiectases of the Skin and Mucous Membranes

**Published:** 1933

**Authors:** Gordon R. Scarff

**Affiliations:** Assistant Surgeon Ear, Nose and Throat Department, Bristol General Hospital


### FAMILIAL MULTIPLE TELANGIECTASES OF THE SKIN AND MUCOUS MEMBRANES.

BY

Gordon R. Scarff, M.B., Ch.B., F.R.C.S.,

*Assistant Surgeon Ear, Nose and Throat Department, Bristol General Hospital.*

---

**Case 1.**—A man, F. S., aged 30, a baker, was sent to see me in April, 1930, complaining of intermittent epistaxis which had started in childhood and which had become more frequent and severe in the last twelve months.

On examination, both nostrils were filled with blood clot, and there was also some purulent discharge present. After removal of this, the nasal mucosa was found to be congested and there were a number of dilated veins present on the anterior part of the septum, extending over a wider area than is usually seen in cases of epistaxis. There was some degree of deflection of the septum to the left, but otherwise nothing further abnormal was noted in the nose or throat, and there was no evidence of accessory sinus infection.

Both sides of the septum were cauterized under local anæsthesia, but despite repetition of this on three occasions during the next six months, the bleeding recurred. He was then admitted to hospital, and a complete general examination made, but no organic disease was found beyond a slight secondary anæmia. The blood picture otherwise showed no abnormality; coagulation time was normal; Wassermann reaction negative. While in hospital he had no further hæmorrhage, and was discharged after five days.

He had no serious recurrence for another twelve months, when he came to see me again. On this occasion it was noticed that he had developed several small angiomata on the skin of the face, the lower lip and the dorsal surface of the tongue, and he stated that his father suffered in the same way. Since that time he has had several small hæmorrhages from the nose, and also slight bleeding from hæmorrhoids. The spots on the face are painless, and vary in size and character, some being simple dilatation of the small vessels of the skin, others having a central dilated vessel surrounded by a stellate network

K

of vessels, while the fully-formed spot is raised above the surface of the skin, and is round or oval in shape, with a sharply-defined margin, varying in colour from bright red to purple. Those present on the lips and tongue are of the last type. He has also lately developed a few small telangiectatic spots under the index and middle finger nails of the right hand.

**Case 2.**—F. S., aged 57, father of the above, was also sent to see me at my request in October, 1931, when I found that he also had multiple angiomata of the face, lips and tongue, and abnormal dilatation of the vessels of the septa mucosa. He had several small telangiectases on both hands situated on the pulp of the fingers and under the finger nails. He had suffered from childhood from recurrent epistaxis, while the other spots had developed since the age of 30. He had marked pyorrhœa, and his gums tended to bleed readily, and he suffered to a slight extent from hæmorrhoids, but beyond this there was nothing abnormal detected.

**Case 3.**—His brother, P. S., aged 63, was also examined, and a similar condition was found.

Blood picture: Wassermann reaction, negative; red cells, 5,210,000 per cub. mm.; hæmoglobin, 76 per cent.; colour index, 0·74; leucocytes, 7,600 per cub. mm.—neutrophile polymorph, 64 per cent.; eosinophile polymorph, 2 per cent.; basophile polymorph, 0 per cent.; lymphocytes, 29 per cent.; large mononuclear, 5 per cent. The film shows normal features. Coagulation time, somewhat increased.

Blood picture, after a moderately severe epistaxis: Wassermann reaction, negative; red cells 4,260,000 per cub. mm.; hæmoglobin, 55 per cent.; colour index, 0·64; leucocytes, 7,600 per cub. mm. Differential count was the same as on the previous occasion. Nothing further of abnormality was noted, except occasional bleeding from hæmorrhoids.

**Case 4.**—A. H., aged 59, a married sister of the above, was referred to me by the late Dr. Carey Coombs two months later on account of recurrent epistaxis, which had started in childhood. She had been sent up for treatment for osteoarthritis of both knee-joints. On examination she was found to have a similar condition of the face, lips, tongue and fingers, but the spots were much fewer in number than in the two previous cases. The septal mucosa was markedly congested, and there was a considerable amount of crusting present. At the time of the examination a slight touch of the septal mucosa was sufficient to start bleeding, but since treatment with a mild antiseptic oil hæmorrhage has been much less easily provoked.

**Cases 5 and 6.**—Since that time I have had referred to me, independently, for epistaxis, a boy aged 8, the son of P. S., and another boy aged 8, grandson of A. H. In neither case was there any marked infection of the tonsils, nose or nasopharynx, and there was no tonsil or adenoid hypertrophy, but there was dilatation of the vessels on the anterior part of the septum, in no way dissimilar from that frequently found in children suffering from nasal obstruction. In addition, the second boy had a small telangiectatic area of the skin over the zygoma on the right side.

**Cases 7 and 8.**—I have also seen a daughter of A. H., G. C., aged 42, and her daughter, aged 17, both of whom suffer from recurrent epistaxis, the mother having several telangiectatic spots on the face, but not elsewhere.

*Clinical Features.*

These patients, and others whom I have not yet seen, are all descendants of one woman, M. S., who died some years ago at the age of 65, and who, it is stated, herself showed angiomatous swellings of her face, lips and tongue, and suffered from recurrent epistaxis.

So far as can be ascertained the condition was not present in either her father or mother. The family tree (see p. 119) shows that up to the present time her descendants number twenty-six, of whom fourteen suffer from recurrent epistaxis, and the nine older members show multiple angiomata (one daughter, who died of influenza aged 40, also showed the condition). It will be seen that both sexes are affected, and that both sexes transmit the tendency, and although in one case a grandson has escaped, it is appearing again in the great-grandson.

The sequence of events has been the same in each case. The child is born healthy, and shows nothing abnormal until the age of 7 or 8 years, when he or she begins to suffer from recurrent epistaxis, the attacks recurring at varying intervals of days, weeks or months. Except in the small boy previously mentioned, no further development appears to take place until about the age of 30, when telangiectatic spots appear on the face, lips and tongue, which increase in size for a time, and after that remain stationary. There is occasional bleeding from the spots on the mucous membrane. Apart from a secondary anæmia after severe epistaxis, the condition does not give rise to any constitutional disturbance.

In the cases examined the blood picture is normal, and in none of the family is there prolonged bleeding after cuts or injuries, nor any tendency to ecchymoses after slight trauma. The male members show a tendency to bleeding from hæmorrhoids. Menstruation in the females is normal. There is no menorrhagia or metrorrhagia. In the case of the married members labours have been normal, and there has been no excessive pre- or post-partum hæmorrhage.

This family is an addition to the number of cases of this rather rare condition which has been already published by various authorities.

The condition was first described by Rendu,[1] of Paris, in 1896, and again by Osler,[2] in America, in 1901, and again in England in 1907.[3] Notes on a third family were published by Parkes-Weber,[4] in 1907. Cases have appeared intermittently in the literature since then, and in 1931 Goldstein[5] published a descriptive summary and bibliography of all the published cases, ninety-five families, involving over five hundred persons. He termed the condition "heredo - familial angiomatosis with recurring hæmorrhages (Rendu-Osler-Weber's disease)." Two further cases have been reported this year by Aubertin, Lévy and Mme. Baclesse.[6]

All the cases reported correspond closely with those of the present series in their main points, viz.:

(*a*) The disease is definitely hereditary and familial.

(*b*) The average proportion of persons affected in each family is one in three, but may be as high as eight in eleven.

(*c*) The condition has been observed in three, four and five generations.

(*d*) The condition occurs in, and is transmitted by, both sexes, although on the whole a slight predominance in females has been observed.

(*e*) There is no family history of syphilis, alcoholism or hepatic disease.

(*f*) In all cases the affection is divided into two stages; the first stage commencing in childhood, and taking the form of recurrent epistaxis; the second stage of angiomata of the face, lips, mouth, tongue and fingers, commencing between the ages of 20 and 30. The angiomata are similar to those described in the present series.

(*g*) In all cases, except for secondary anæmia, the blood picture is normal. There is no excessive fragility of the red corpuscles. The number of blood platelets and the amount of blood calcium is normal. Coagulation time and bleeding time occasionally show delay, but only to a very slight extent. There is no purpuric condition present.

Although in the majority of cases these are the only manifestations, further symptoms have been observed in some cases, viz.:—

(i.) The condition may be present on other parts of the body, especially on the fore-arms and hands.

(ii.) Hæmoptysis has occurred in some cases from the larynx or bronchi, and a case of fatal hæmorrhage from the larynx has been recorded.

(iii.) Hæmatemesis has occurred with no sign or symptom of gastric ulcer, and telangiectases have been found in the mucosa of the stomach at autopsy.

(iv.) Hæmorrhages may occur from the rectum and anal canal.

(v.) Varicose veins are a frequent occurrence in the later stages of the disease.

(vi.) Metrorrhagia is occasionally present.

(vii.) Some enlargement of the liver or spleen, or both, has been noted in a few cases, but in these only occurs in the late stages after prolonged secondary anæmia due to frequent severe hæmorrhages.

*Pathology.*

In cases where sections have been cut at autopsy the structure of the spots is found to be a simple blood space immediately under the epithelium, lined by endothelium, and supported by a small amount of connective tissue. There are no supporting muscle or elastic fibres, and the epithelium over the space is thinned but otherwise normal. Weil[7] has put forward the view that the condition is due to either a primary blood or hepatic dyscrasia. Evidence, however, against this view is supplied by the fact that in the cases described, and in practically all others reported, no abnormality of the blood has been found; nor in the present series has there been any clinical evidence of hepatic or splenic enlargement. Moreover, in the cases of this series where the Van den Bergh reaction has been performed this has shown a completely negative result, that is, no abnormal hæmolysis is taking place in the blood-stream. The

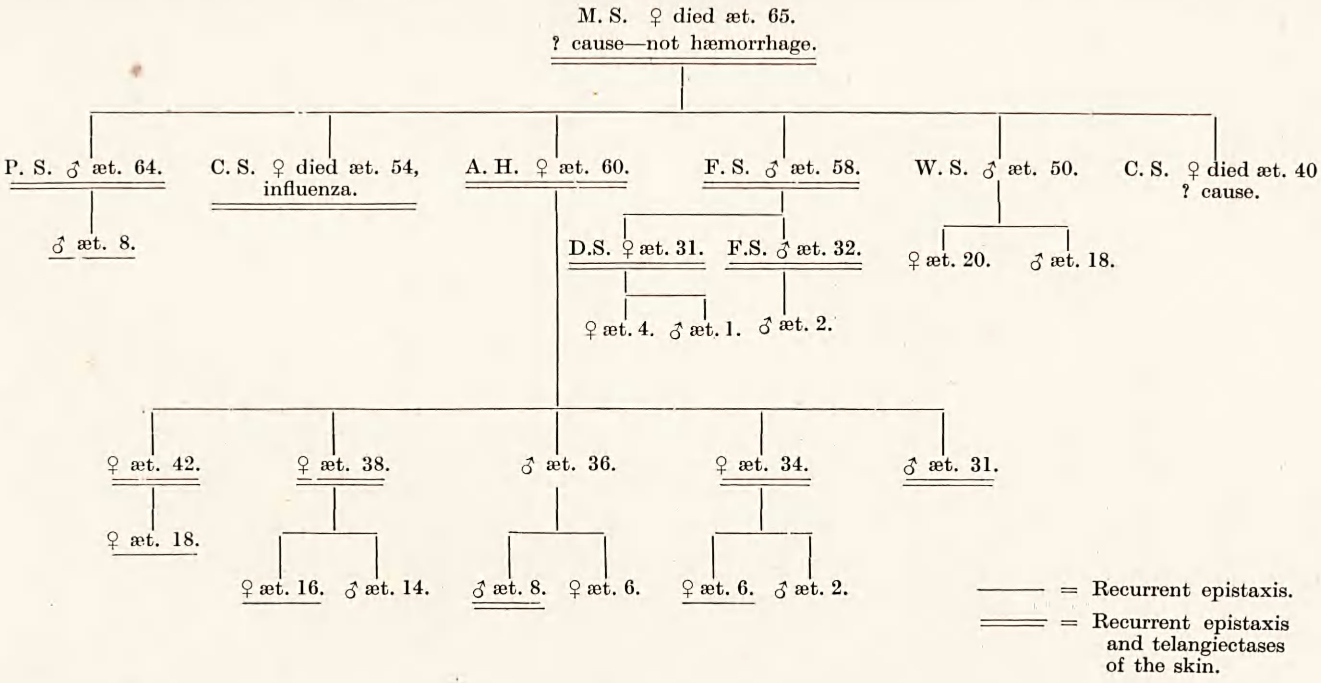

M. S. ♀ died æt. 65.

? cause—not hæmorrhage.

P. S. ♂ æt. 64.

C. S. ♀ died æt. 54, influenza.

A. H. ♀ æt. 60.

F. S. ♂ æt. 58.

W. S. ♂ æt. 50.

C. S. ♀ died æt. 40 ? cause.

♂ æt. 8.

D.S. ♀ æt. 31.

F.S. ♂ æt. 32.

♀ æt. 20.

♂ æt. 18.

♀ æt. 4.

♂ æt. 1.

♂ æt. 2.

♀ æt. 42.

♀ æt. 38.

♂ æt. 36.

♀ æt. 34.

♂ æt. 31.

♀ æt. 18.

♀ æt. 16.

♂ æt. 14.

♂ æt. 8.

♀ æt. 6.

♀ æt. 6.

♂ æt. 2.

= Recurrent epistaxis.

= Recurrent epistaxis and telangiectases of the skin.

condition would appear to be simply a congenital weakness of the walls of the smaller vessels with deficiency in muscle and elastic tissue support.

*Diagnosis.*

In childhood the cause of the epistaxis can only be ascertained by inquiry into the family history. In adults the appearance of the spots and the normal blood picture serve to distinguish the condition from one of hæmophilia, purpura or multiple nævi.

*Prognosis.*

The condition appears to be compatible with fairly healthy life, and does not seem to shorten life to any great extent. Of the recorded cases only five have died from hæmorrhage.

*Treatment.*

This is limited to treatment of the secondary anæmia and to treatment of the epistaxis when it occurs. Prolonged treatment by a mild antiseptic nasal oil, by keeping the nasal mucosa in a healthy state, appears to lessen the number of hæmorrhages, but application of the cautery does not seem to prevent recurrence in another situation.

My thanks are due to Dr. Hassard and Dr. Milling for their help in tracing different branches of the family.

REFERENCES.

[1] Rendu, *Gazette des Hôpitaux de Paris*, No. 135, 1896, p. 1,332.

[2] Osler, *Bulletin of Johns Hopkins Hospital*, November, 1901, p. 333.

[3] Osler, *Quarterly Journal of Medicine*, 1907, i. 53.

[4] Parkes-Weber, *Lancet*, 1907, ii. 160.

[5] Goldstein, *Archives of Internal Medicine*, 1931, xlviii. 836.

[6] Aubertin, Lévy and Mme. Baclesse, *La Presse Médicale*, 1933, p. 185.

[7] P. E. Weil, *Le Sang*, 1927, i. 35; Weil and Isch-Wall, *Soc. Méd. Hôp. Paris*, 11th July, 1930, p. 1,278.

